# A Comprehensive Analysis of the Intrinsic Visible Fluorescence Emitted by Peptide/Protein Amyloid-like Assemblies

**DOI:** 10.3390/ijms24098372

**Published:** 2023-05-06

**Authors:** Nicole Balasco, Carlo Diaferia, Elisabetta Rosa, Alessandra Monti, Menotti Ruvo, Nunzianna Doti, Luigi Vitagliano

**Affiliations:** 1 Institute of Molecular Biology and Pathology, National Research Council (CNR), Department of Chemistry, University of Rome Sapienza, Piazzale Aldo Moro 5, 00185 Rome, Italy; nicole.balasco@cnr.it; 2 Department of Pharmacy and CIRPeB, Research Centre on Bioactive Peptides “Carlo Pedone”, University of Naples “Federico II”, Via Montesano 49, 80131 Naples, Italy; carlo.diaferia@unina.it (C.D.); elisabetta.rosa@unina.it (E.R.); 3 Institute of Biostructures and Bioimaging (IBB), National Research Council (CNR), 80131 Napoli, Italy; alessandra.monti@ibb.cnr.it (A.M.); menotti.ruvo@unina.it (M.R.)

**Keywords:** protein and peptide self-assembling, optical properties of amyloids, fluorescence, photoluminescence, bioinspired materials, supramolecular chemistry, bioimaging

## Abstract

Amyloid aggregation is a widespread process that involves proteins and peptides with different molecular complexity and amino acid composition. The structural motif (cross-β) underlying this supramolecular organization generates aggregates endowed with special mechanical and spectroscopic properties with huge implications in biomedical and technological fields, including emerging precision medicine. The puzzling ability of these assemblies to emit intrinsic and label-free fluorescence in regions of the electromagnetic spectrum, such as visible and even infrared, usually considered to be forbidden in the polypeptide chain, has attracted interest for its many implications in both basic and applied science. Despite the interest in this phenomenon, the physical basis of its origin is still poorly understood. To gain a global view of the available information on this phenomenon, we here provide an exhaustive survey of the current literature in which original data on this fluorescence have been reported. The emitting systems have been classified in terms of their molecular complexity, amino acid composition, and physical state. Information about the wavelength of the radiation used for the excitation as well as the emission range/peak has also been retrieved. The data collected here provide a picture of the complexity of this multifaceted phenomenon that could be helpful for future studies aimed at defining its structural and electronic basis and/or stimulating new applications.

## 1. Introduction

**Figure 1:**
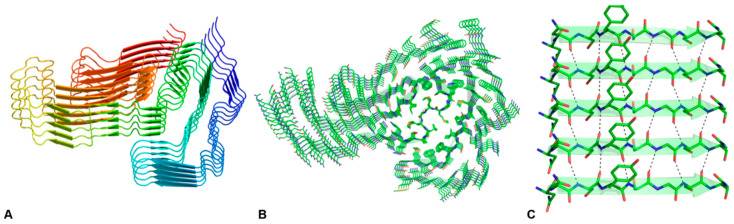
Lateral (**A**) and top (**B**) view of amyloid fibrils formed by the low-complexity domain of TDP-43 (TDP-43 LCD) in the cryo-EM structure (PDB code 7KWZ). In Panel (**A**), ribbons are represented with a color code (blue to red) from the N- to the C-terminus. The model has a similar orientation in Panel (**B**). The H-bonding pattern of the parallel β-sheet formed by residues 371–378 is shown in Panel (**C**), in which the N-terminal side of the chain is on the left.

Proteins are biological macromolecules that play major roles in all physio-pathological processes observed in living organisms. Classically, the functionality of these complex biomolecules has been associated with their ability to adopt one or a discrete number of well-defined (folded) states that present a slight, but fundamental, thermodynamic over-stabilization compared to the ensemble of highly flexible structures that characterize their unfolded status. This delicate balance between folded and highly flexible states was considered to be fundamental for the functional regulation of these proteins. Studies carried out in recent decades have clarified that this scenario is a non-trivial over-simplification of the protein behavior. Indeed, a significant portion of proteins operate without adopting well-defined structures but they rather function via the ensembles of flexible states (intrinsically disordered proteins) [1]. Moreover, on the other hand, it has been discovered that along with their chameleonic behavior, proteins may also assume, starting either from folded or unfolded states, highly rigid and stable structures (protein misfolding) [2]. These structures, also denoted as amyloids, may be either soluble or insoluble and are characterized by a large content of β-structure with the peptide chains running perpendicular to the elongation axis of these assemblies (cross-β structure) [3] (see Figure 1 for a representative example). Interestingly, these structural motifs have also been detected in the functional forms of proteins and peptides. Further investigations carried out on cross-β structures have unraveled the unexpected and widespread tendency of polypeptide chains, characterized by remarkable diversity in terms of size, chemical composition, and polarity, to adopt this state. The propensity of the polypeptide chain to adopt these amyloid-like states that are endowed with several structural and functional properties has also raised the suggestion that they may be eventually involved in the origin of life [4].

In addition to the well-established role of amyloids in many physio-pathological processes, the discovery that even small peptides may undergo self-assembling with the formation of cross-β structures has also stimulated studies on these systems in other fields. In particular, peptides and proteins that can self-assemble as amyloids represent valuable tools for the design and the advent of innovative biomaterials with a wide range of applications in biomedicine and material sciences [5,6,7,8]. Indeed, the peculiar structural features of amyloids have been profitably exploited to obtain materials with special mechanical properties. The network of hydrogen bonds that stabilize the β-structure of the cross-β motif has been demonstrated to be a well-suited basic element of physical hydrogels.

**Figure 2:**
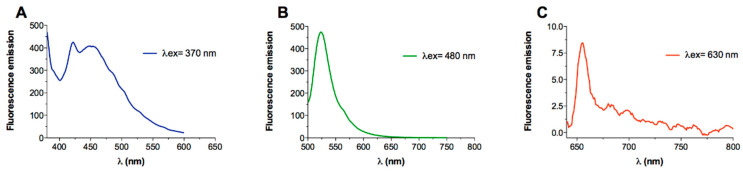
Representative fluorescence emissions in visible regions of the spectra by peptide amyloid-like assemblies. The spectrum with emission in the blue region has been registered on the PEG8-F6 peptide (Panel (**A**)), whereas those with green and red emission (Panels (**B**,**C**)) have been collected on PREP1. These peptides are described in the text and tables.

Interestingly, the association of peptide/protein chains in the cross-β motif generates aggregates whose special properties go beyond the mechanical ones. Indeed, the characterization of these amyloid aggregates has demonstrated that they are endowed with interesting and unexpected spectroscopic properties. In particular, it has been shown that these assemblies can emit intrinsic label-free fluorescence radiation in the regions of the electromagnetic spectrum that are not associated with the typical emission of proteins and peptides. Indeed, after some sporadic but intriguing observations reported in the first decade of the century, studies carried out in the last decade have demonstrated the ability of amyloid assemblies and aggregates to emit visible fluorescence independently of their physical state (solid, liquid, or hydrogel) and amino acid composition. In particular, it has been well assessed that the presence of aromatic residues in their sequence is unessential for generating this fluorescence [9]. Moreover, although this information is somehow overlooked, recent and current studies are progressively showing the complexity of this visible fluorescence, as selective emissions in different wavelength ranges can be stimulated. Indeed, it has been demonstrated that some specific amyloids may produce multicolor fluorescence with the maximum of the emission located in the blue, green, red, and even in the infrared region of the electromagnetic spectrum (see Figure 2).

It is important to note, however, that, despite the progressive interest in this peculiar phenomenon and its many implications in both basic and applied science, its underlying structural and electronic basis is yet to be unraveled. Although different hypotheses have been proposed, the topic is still highly debated and no consensus has been hitherto achieved.

In recent years, several insightful reviews have focused on different aspects of fluorescence emission by amyloids. In particular, these studies have addressed both basic aspects of this puzzling phenomenon as well as promising applications [9,10,11,12,13,14,15,16,17,18,19,20,21,22,23,24,25,26]. Nevertheless, systematic surveys of amyloid systems that can emit visible fluorescence and their spectroscopic properties have not yet been provided. An exhaustive analysis of the literature reporting the fluorescence emission of proteins and peptides in the amyloid state could represent a useful source of information for studies aimed at identifying the elusive structural basis of this intriguing phenomenon and stimulating new applications. We here report a systematic analysis of the papers reporting original data on this topic. Interestingly, as the discovery of the visible fluorescence by amyloids has stimulated similar studies on self-assembling systems with a smaller size, such as individual amino acids, here, we also survey the visible fluorescence of these compounds, as they could share a common underlying mechanism. To provide quantitative information on this puzzling phenomenon, in vitro studies represent the main focus of the manuscript, although in vivo applications will also be mentioned.

## 2. Exhaustive Search of Literature Articles Describing the Visible Fluorescence Emission by Amyloid-like Systems

Papers describing intrinsic visible fluorescence emission by amyloid-like systems were identified using multiple PUBMED and Google Scholar searches (February 2023). The papers identified in these searches were individually inspected to check their relevance to the topic of the review.

In particular, considering that intrinsic fluorescence has been identified in the literature using different notations (label-free fluorescence, intrinsic fluorescence, photoluminescence, etc.), several distinct PUBMED searches were performed (Table S1). From this initial survey, we selected 87 manuscripts of potential interest. Due to the heterogeneity in the designation of this fluorescence, we integrated these PUBMED searches with Google Scholar surveys looking at the 185 documents citing one of the pioneering and seminal papers on this subject [27]. This led to the identification of 213 novel manuscripts of interest.

All of the 213 selected papers were individually inspected selecting only original reports of fluorescent emission by amyloid-like systems. This further selection meant that we ended up with 92 papers, and from which the information described in the present manuscript was retrieved. In particular, from each paper, the following data were collected: chemical entity of the emitting system (name and UniProt ID for proteins and sequence for peptides), excitation and emission wavelength range, methodology used for the experiment, the physical state of the sample, and its size.

## 3. Amyloid-like Assemblies Exhibiting Intrinsic Visible Fluorescence

Once identified, the amyloid systems emitting visible fluorescence radiation were grouped and classified as a function of their molecular complexity. Although the ability of small peptides, and even amino acids, to emit visible fluorescence was first reported two decades ago [28,29], the correlation between the aggregation of polypeptide chains and special spectroscopy properties has been mainly highlighted via the characterization of amyloids formed by proteins involved in neurodegenerative diseases. Reductionist approaches have then been used to extend these findings to small peptides. Therefore, in the following sections and tables, their properties will be described starting with proteins. Then, peptides, both linear and cyclic, will be considered. Finally, the spectroscopic properties of individual amino acids will be illustrated.

### 3.1. Proteins

It is well known that proteins may undergo amyloid-like association independently of their initial functional structure. Even proteins whose native structure contains only α-helices may form β-rich amyloid structures. The prototypal example in this context is represented by myoglobin, whose ability to form aggregates in slightly denaturing conditions was discovered twenty years ago [30,31]. On the other hand, although the sequences of intrinsically disordered proteins seem to be specifically designed to prevent aggregation, they can also form amyloids.

An inspection of Table 1 clearly shows that intrinsic visible fluorescence has been frequently detected in amyloids formed by proteins. Indeed, it has been demonstrated that more than thirty unrelated proteins are endowed with this peculiar property. Notably, this radiation is emitted in a variety of different physical states, which include solution, crystalline, liquid crystals, fibrillar, suspensions, and amorphous states.

Most of these proteins emit fluorescence in the blue region of the visible spectrum (420–470 nm) upon excitation in the wavelength range of ~320–380 nm. Green fluorescence, with a wavelength of the maximum typically centered at ~520 nm and observed with excitation in the range of ~400–480 nm, has been reported for the HET-s prion domain, α-synuclein, α-lactalbumin, β-lactoglobulin, insulin, human/bovine serum albumin, K18 tau, GRASP ancestor 4, and elastin. Among these, elastin presents peculiar behavior since the excitation at 325 nm induces fluorescence emission in 2 distinct regions of the spectrum with maxima at 415 (blue) and 505 (green) nm. The authors interpreted these data by assuming that the 415 and 505 nm peaks, whose intensities increase upon aging and likely aggregation, could be generated via aromatic residues and their interaction, respectively (N43). However, these findings may be alternatively interpreted by assuming that the emission in the blue region is the radiation generated by the association of β-rich structures, whereas the green fluorescence could have originated, as a sort of RET (resonance electron transfer), via the absorption of the blue radiation.

Importantly, it has been recently reported that the emission spectra of protein amyloids can extend up to the red and the near infra-red (NIR) regions [32]. Although the intensity of this radiation is much lower than that measured for the blue emission, clear red/NIR fluorescence has been detected for the HET-s prion domain, insulin, lysozyme, α-synuclein, α-lactalbumin, β-lactoglobulin, human serum albumin, and transthyretin (Table 1).

**Figure 3:**
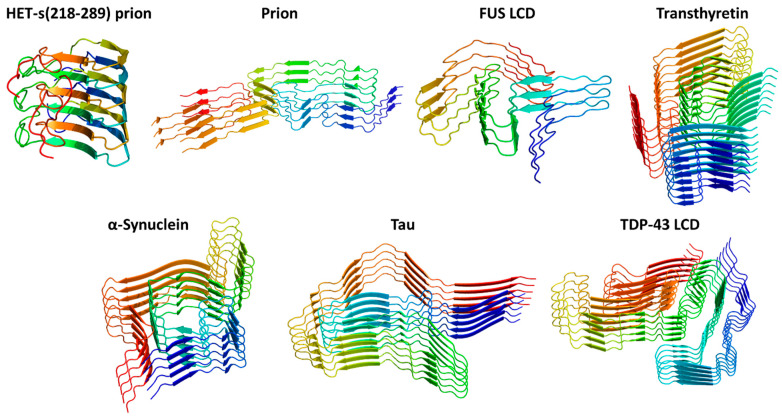
Representative examples of proteins adopting cross-β structures determined at the atomic level. The PDB codes of the structures reported are 2KJ3 (HET-s(218–289) prion), 7LNA (Prion), 7VQQ (FUS low-complexity domain LCD), 7OB4 (Transthyretin), 6L1T (α-Synuclein), 7P6D (Tau), and 7KWZ (TDP-43 low-complexity domain LCD). Ribbons are represented with a color code (blue to red) from the N- to the C-terminus.

One of the most interesting results that emerged from the spectroscopic characterization of protein amyloids is the observation that the wavelength of the emitted fluorescence may systematically depend on the wavelength of the radiation used for the excitation. Indeed, the characterization of amyloid aggregates formed by insulin and β-lactoglobulin indicates that the increment of the excitation wavelength in the 310–450 nm range produces an emission with an increasing wavelength (maxima located in the range of ~410–510 nm) [33]. This observation represents a violation of Kasha’s rule, which states that the emission of a photon in a molecular system is generated by the decay from the lowest excited state. The data observed for insulin and β-lactoglobulin were interpreted by the authors by invoking the red edge excitation shift (REES) phenomenon [34], which is generated via solvent–fluorophore interactions. If the relaxation of the solvent is faster than the fluorescence decay, a redshift of the emission wavelength, independent of the excitation, is observed. In highly rigid environments, such as those occurring in amyloid aggregates, the tight association of the molecules makes the relaxation of the solvent molecules, if present, particularly long, and high-energy emissions can also be detected; thus, a wavelength-dependent fluorescence emission is observed. Similar effects have also been observed for amyloids formed by peptides, even in solution states [35,36]. Although the three-dimensional structures of some of these amyloids have been reported in the Protein Data Bank (PDB) (see Figure 3 for representative examples), caution should be taken in deriving correlations between the structure and the spectroscopic properties. Indeed, structural studies carried out in the last decade on amyloids formed by proteins suggest that a single chain may adopt different structures (polymorphism). Unfortunately, structural studies that have been successful in recent years, especially via the application of cryo-electron microscopy, are generally not complemented with spectroscopic characterizations. Therefore, specific spectroscopic features cannot be safely associated with a specific structural polymorph.

**Table 1:** Intrinsic visible fluorescence emission detected in amyloids formed by proteins.

Protein System	UniProt ID	Excitation, Emission Wavelength/Range (λexc, λem)			Methodology Used for the Experiment	Physical State of the Sample	Ref.
		UV–Blue	Green	Red–NIR			
Gamma-B (Gamma-II) Crystallin	P02526	340 nm, 445 nm			Fluorescence spectroscopy	Aggregates	[37]
		340 nm, 425/445 nm			Fluorescence spectroscopy	Protein solution	
		354/361 nm, 466 nm			Fluorescence spectroscopy	Protein solution	[38]
Triosephosphate Isomerase (TIM)	P62002	351/364 nm, 445 nm			Fluorescence spectroscopy, microscopy	Needle-like crystals	[37]
Truncated TIM (β_1_α_1_β_2_ domain with Y and F but no W residues)		340 nm, 425 nm			Fluorescence spectroscopy	Protein solution	
Hydantoinase	Q5DLU2	351/364 nm, 450 nm			Fluorescence spectroscopy	Crystals	[37]
Hen egg white lysozyme (HEWL)	P00698	351/364 nm, 470 nm			Fluorescence spectroscopy	Crystalline form	[37]
		354/361 nm, 425 nm			Fluorescence spectroscopy	Protein solution	[38]
		355 nm, 440 nm			Fluorescence spectroscopy	Amyloid fiber suspension	[27]
		357 nm, 440 */470 * nm			Fluorescence spectroscopy	Amyloid fiber suspension	[39]
		350 nm, 438 nm			Fluorescence spectroscopy	Fibers	[40]
		360 nm, 440 * nm		640 nm, 703 nm	Fluorescence spectroscopy	Amyloid fiber	[32]
		375 nm, 428 nm			Fluorescence spectroscopy	Amyloid fiber suspension	[41]
		365 nm, 445/50 nm			Fluorescence microscopy	Solid state	[42]
		357 nm, 430–450 * nm			Steady-state fluorescence, fluorescence spectroscopy	Amyloid fiber suspension	[42]
		370 nm, 460 * nm			Fluorescence spectroscopy	Protein solution	[43]
		365 nm, 450 nm			Fluorescence spectroscopy	Aqueous concentrates and solid state	[44]
α-Synuclein (α-Syn)	P37840	380 nm, 480 nm	450 nm, 520 nm		Fluorescence spectroscopy	Protein solution	[45]
		405 nm, 460 * nm			Confocal fluorescence lifetime microscopy	Fibrils in solid state	[46]
		405 nm, 450–500 * nm			Confocal microscopy	Fibrils in solid state	[47]
		380 nm, 425 nm			Fluorescence spectroscopy	Amyloid fiber suspension	[48]
Human Lysozyme (I59T mutant)	P61626		450 nm, >488 * nm		Time-correlated single-photon-counting fluorescence lifetime imaging	Fibrils in solid state	[27]
			405 nm, >488 * nm		Confocal microscopy	Fibrils in solid state	
TAR-DNA-binding protein-43 (TDP-43) (prion-like domain, residues 342–414)	Q13148	375 nm, 427–450 nm			Fluorescence spectroscopy	Protein solution	[49]
TDP-43 mutant (prion-like domain, residues 363–414, A315E, and Q331K, and M337V mutations)		375 nm, 446 * nm			Fluorescence spectroscopy	Protein solution	[50]
RNA-binding protein FUS (prion-like domain, residues 1–165)	P35637	375 nm, 421–450 nm			Fluorescence spectroscopy	Protein solution	[49]
RNA-binding protein FUS (RNA-recognition motif domain RRM, residues 285–371)		375 nm, 440 nm			Fluorescence spectroscopy	Protein solution	
Bovine insulin	P01317	310–380 nm, 360–430 * nm	400–420 nm, 430–500 * nm		Fluorescence spectroscopy	Native protein in solution, fibrils and spherulites in solution	[33]
		360 nm, 430 * nm		640 nm, 696 nm	Fluorescence spectroscopy	Amyloid fiber suspension	[32]
		375 nm, 428 nm			Fluorescence spectroscopy	Amyloid fiber suspension	[41]
		375 nm, 425 nm			Fluorescence spectroscopy	Spherulites	[51]
	N.R. **	330–590 nm, 440–610 nm			Fluorescence spectroscopy	Solution of dots	[52]
	N.R. **	350 nm, 438 nm			Fluorescence spectroscopy	Fibers	[40]
	P01308	350 nm, 440 * nm			Fluorescence spectroscopy	Amyloid fiber suspension	[13]
Bovine β-Lactoglobulin (β-LG)	P02754			640 nm, 702 nm	Fluorescence spectroscopy	Amyloid fiber suspension	[32]
		360 nm, 430 nm			Fluorescence spectroscopy	Amyloid fiber suspension	[48]
		365 nm, 420 nm			Fluorescence microscopy, confocal imaging	Hybrid fibers	[53]
		405 nm, 480 * nm	514 nm, 570 * nm	633 nm, 650 * nm	Fluorescence microscopy and spectroscopy	Hybrid tactoids	[54]
		320–380 nm, 360–425 * nm	400–450 nm, 450–525 * nm		Fluorescence spectroscopy	Native protein in solution, fibrils and spherulites in solution	[33]
HET-s prion-domain (residues 218–289)	Q03689	360 nm, 450 nm	440 nm, 520 * nm	640 nm, 700 * nm	Fluorescence spectroscopy	Amyloid fiber suspension	[32]
		390 ± 10 nm, 460 ± 50 nm	475 ± 10 nm, 530 ± 50 nm	620 ± 60 nm, 700 ± 75 nm	Fluorescence microscopy	Highly oriented amyloid fibers	
Prion C-terminal fragment (MoPrP (89–230))	P04925	375 nm, 428 nm			Fluorescence spectroscopy	Amyloid fiber suspension	[41]
Human serum albumin (HSA)	P02768	375 nm, 450 * nm	460 nm, 535 * nm		Fluorescence spectroscopy	Protein in solution	[55]
		405 nm, 450 * nm			Confocal microscopy	Fibrils in solid state	
				640 nm, 696 nm	Fluorescence spectroscopy	Amyloid fiber suspension	[32]
Bovine serum albumin (BSA)	P02769	355 nm, 425 nm			Fluorescence spectroscopy	Protein solution	[56]
		320 nm, 420 nm 365 nm, 448 nm	440 nm, 500 nm		Fluorescence spectroscopy	Protein solution and solid state	[44]
Human Tau isoform K18 (K18Tau I260C/C291A/C322A) variant (129 residues)	P10636	405 nm, 460 * nm			Confocal fluorescence lifetime images	Fibrils in solid state	[46]
			450 nm, >480 * nm		Time-correlated single-photon-counting fluorescence lifetime imaging	Fibrils in solid state	[27]
			405 nm, >488 * nm		Confocal microscopy	Fibrils in solid state	
Myoglobin (MB)	P68082	405 nm, 470 * nm			Fluorescence spectroscopy	Protein solution	[57]
		405 nm, 450–500 * nm			Confocal microscopy	Solid state	
Transthyretin (TTR) V30M mutant	P02766			640 nm, 695 nm	Fluorescence spectroscopy	Amyloid fiber suspension	[32]
α-Lactalbumin	P00711			640 nm, 692 nm	Fluorescence spectroscopy	Amyloid fiber suspension	[32]
Human insulin	P01308	275nm, 440 * nm			Fluorescence spectroscopy	Protein solution	[58]
Keratin-based films	N.R.	280 nm, 450 * nm			Fluorescence spectroscopy	Nanorods in solution	[59]
Polyclonal Rabbit Immunoglobulin (rIgG)	N.R.	300–320 nm, 400–450 nm			Fluorescence spectroscopy	Protein solution	[60]
GRASP55	Q9H8Y8	375 nm, 440 nm			Fluorescence lifetime imaging microscopy	Fibrils in solid state	[61]
GADD45α, GADD45β	P24522, O75293	360 nm, 420/455 nm			Fluorescence spectroscopy	Protein solution	[62]
Elastin	P04985	325 nm, 415 nm	325 nm, 505 nm		Fluorescence spectroscopy	Protein solution	[63]
Human transthyretin (TTR)	P02766	360 nm, 417/438/480 nm			Fluorescence spectroscopy	Protein solution	[64]
		360 nm, 400/420 nm, and 360 nm, 417/438 nm			Fluorescence spectroscopy	Protein solution	[65]
Double Plant homeodomain fingers 3 isoform a (DPF3a)	Q92784	350 nm, 460 * nm			Fluorescence spectroscopy	Amyloid fiber suspension	[66]
DPF3a and DPF3b	Q92784	400 nm, 456 nm			Fluorescence spectroscopy	Amyloid fiber suspension	[67]
DPF3a (residues 200–357) and DPF3b (residues 200–378)	Q92784	400 nm, 460 nm			Fluorescence spectroscopy	Amyloid fiber suspension	[68]
Class I hydrophobin Vmh2 and Vmh2-H3w chimera (H3w fluorescent peptide HPHGHW)	Q8WZI2	345 nm, 450 nm			Fluorescence spectroscopy	Protein solution	[69]
TasA	P54507	350 nm, 435 nm			Fluorescence spectroscopy	Amyloid fiber suspension, dry samples	[48]
GRASP ancestors ANC1, ANC2, ANC3, and ANC4	N.R. **	365 nm, 450 * nm	365 nm, 510 * nm (for ANC4)		Fluorescence spectroscopy	Protein solution	[70]

* Data inferred from the figures of the related reference. ** Not reported (N.R.).

### 3.2. Peptides

The analysis of the reports in the literature indicates that several peptides or peptide-containing systems are endowed with the ability to emit fluorescence in the visible region of the spectrum. In the following paragraphs, we initially describe the fluorescence emission of linear peptides that can form standard cross-β motifs. We then illustrate the spectroscopic properties of assemblies formed by cyclic dipeptides, which, although they cannot form the canonical hydrogen-bonding patterns that stabilize β-sheets, may originate from supramolecular associations endowed with the ability to emit fluorescence. Finally, we survey the spectroscopic properties of individual amino acids, PNA, and PNA–peptide conjugates.

#### 3.2.1. Linear Peptides

The ability of self-assembled peptides to generate visible fluorescence was reported nearly 20 years ago when Shukla et al. [37] described this peculiar spectroscopic property for the peptide with sequence NDSIRSCRLIPQHT, which does not present any aromatic fluorescent residue in its sequence. The ability to emit fluorescence in the visible region by self-assembled systems made of peptide chains composed of non-aromatic residues was confirmed by the characterization of fibrils formed by poly(VGGLG) [71]. Structural and spectroscopic investigations on self-assembled peptides have been significantly stimulated via the discovery that the dissection of aggregating proteins could generate peptide fragments forming different supramolecular assemblies, including amyloids. As reported in Table 2, fluorescent peptide aggregates were derived from several proteins, including γ-crystallin, human serum albumin, nucleophosmin, β-lactoglobulin, transthyretin, elastin, and the homeobox protein PKNOX1 (also denoted as PREP1). Similarly, self-assembling fluorescent peptides were also obtained via the fragmentation of polypeptides such as Aβ(1–42) or islet amyloid polypeptide human amylin (hIAPP). These peptides present a large spectrum of variability in terms of size, amino acid composition, and polarity. Interestingly, fluorescent amyloids are also formed by linear dipeptides. Again, the presence of aromatic residues is not essential for fluorescence emission. In this context, it is interesting to note that the emission spectra of a peptide derived from the protein PREP1 (residues 117–132) that does not contain any aromatic residue show a signal corresponding to its intrinsic green fluorescence, which is also detectable in the presence of the Thioflavin (ThT) dye [72,73]. Indeed, the presence of the peak intensity at 520 and not at 480 nm, the wavelengths typically observed for ThT, demonstrates that the intrinsic fluorescence of this peptide overcomes that produced by the amyloid-bound dye. The remarkable intensity of the fluorescence emitted by this peptide has also been observed in other very different contexts, such as solid-state and living cells. We hypothesize that the double peak (at ~480 and 520 nm) detected for the ThT-treated zinc finger protein DPF3 (Double PHD fingers 3) is ascribed to the dye and intrinsic green fluorescence, respectively [67].

Despite the obvious interest in peptides containing only polar and aliphatic residues [9,74], the design and the development of peptides formed by aromatic residues, which present a special propensity for aggregation, have provided insightful information on this puzzling phenomenon. In particular, starting from the characterization of the diphenylalanine dipeptide and its many variants, the characterization of peptides containing Phe residues has highlighted the complexity of this peculiar fluorescence. In particular, the characterization of the hexa-phenylalanine peptide (F6) conjugated to different types of polyethylene glycol (PEG) moieties has clearly demonstrated that the spectroscopic properties of the adduct depend on the chemical properties of the PEG. Indeed, while these PEG-F6 derivatives generally present blue fluorescence [75,76], the one presenting the longest PEG chain (PEG24-F6) shows an additional green emission at 530 nm [76]. Although the origin of this observation remains obscure, it can be speculated that different PEG moieties may differently affect the fine details of the nanostructure of these assemblies, which, in turn, is reflected in different spectroscopic properties. Notably, the ability of PEG-F6 films to generate, in addition to the blue, green fluorescent bands has been considered to be promising evidence for the generation of biocompatible tunable fluorescent-light-delivering probes in light diagnostics and precision medicine.

The spectroscopic properties of phenylalanine-based peptides have been used to evaluate the relative strength of the forces that favor amyloid-like associations and self-assembling based on the Watson–Crick pairing in conjugates formed by the FF dipeptide and peptide nucleic acids/nucleobases (see Table 3). These investigations have demonstrated the forces underlying the amyloid-like association which overcame those generated by the base pairing in dictating the organization of these supramolecular assemblies [77].

**Table 2:** Intrinsic visible fluorescence emission detected in amyloids formed by peptides. Amino acids are reported using one-letter code. D-amino acids are indicated using lower-case letters.

Peptide System	Peptide Sequence	Excitation and Emission Wavelength Range (λexc, λem)			Methodology Used for the Experiment	Physical State of the Sample	Ref.
		UV–Blue	Green	Red–NIR			
IAPP-derived peptide	CSNNFGA			640 nm, 695 nm	Fluorescence spectroscopy	Amyloid fiber suspension	[32]
Poly(VGGLG)	VGGLG	405 nm, 465 nm			Confocal microscopy	Amyloid fiber suspension	[71]
TTR-derived peptide (residues 105–115)	YTIAALLSPYS	360 nm, 440 nm			Fluorescence spectroscopy	Amyloid fiber suspension	[78]
Elastin-derived peptide	GVGVAGVG	358 nm, 460 nm			Fluorescence spectroscopy and microscopy	Film	[79]
Gamma-B (Gamma-II) crystallin-derived peptide lacking aromatic residues	NDSIRSCRLIPQHT	345 nm, 425 nm			Fluorescence spectroscopy	Lyophilized (powder) form	[37]
		340 nm, 415–420 nm			Fluorescence spectroscopy	Protein solution	
HSA-derived peptide	FLSucFF	375 nm, 450–460 * nm			Fluorescence spectroscopy	Protein solution	[55]
HSA-derived peptide	WLSucLW	375 nm, 450–460 * nm			Fluorescence spectroscopy	Protein solution	
HSA-derived peptide lacking aromatic residues	LLSucLL	375 nm, 450–460 * nm			Fluorescence spectroscopy	Protein solution	
Homeobox protein PKNOX1-derived peptide (residues 117–132)	LMVKAIQVLRIHLLEL	370 nm, 420–450 * nm	400–480 nm, 520 * nm		Fluorescence spectroscopy	Amyloid fiber suspension	[72]
		405 ± 10 nm, 460 ± 50 nm	475 ± 10 nm, 530 ± 50 nm	620 ± 60 nm, 700 ± 75 nm	Fluorescence microscopy	Cells and fibrils in solid state	
Homeobox protein PKNOX1-derived peptide (residues 117–132)	R8βAlaLMVKAIQVLRIHLLE	370 nm, 420–450 * nm	488 nm, 520 * nm	555 nm, 710 nm	Fluorescence spectroscopy	Amyloid fiber suspension	[72]
		390 ±10 nm, 460 ± 50 nm	475 ± 10 nm, 530 ± 50 nm	620 ± 60 nm, 700 ± 75 nm	Fluorescence microscopy	Cells and fibrils in solid state	
Homeobox protein PKNOX1-derived peptide (residues 297–311)	R8βAlaAQTNLTLLQVNNWFI	370 nm, 420–450 * nm	400–480 nm, 520 * nm	670 nm, 710 nm	Fluorescence spectroscopy	Amyloid fiber suspension	[72]
		390 ± 10 nm, 460 ± 50 nm	475 ± 10 nm, 530 ± 50 nm	620 ± 60 nm, 700 ± 75 nm	Fluorescence microscopy	Cells and fibrils in solid state	
β-Amyloid peptide (1–42) (Aβ(1–42))	DAEFRHDSGYEVHHQKLVFFAEDVGSNKGAIIGLMVGGVVIA	360 nm, 440 * nm	440 nm, 520 * nm	640 nm, 696 nm	Fluorescence spectroscopy	Amyloid fiber suspension	[32]
		390 ± 10 nm, 460 ± 50 nm	475 ± 10 nm, 530 ± 50 nm	620 ± 60 nm, 700 ± 75 nm	Fluorescence microscopy	Amyloid fiber suspension	
		405 nm, 460 * nm			Confocal fluorescence lifetime microscopy	Fibrils in solid state	[46]
		390 nm, 430 * nm			Fluorescence spectroscopy	Fibrils in solution	[80]
			450 nm, >488 * nm		Time-correlated single-photon-counting fluorescence lifetime imaging	Fibrils in solid state	[27]
			405 nm, >488 * nm		Confocal microscopy	Fibrils in solid state	
		365 nm, 470 nm			Fluorescence spectroscopy	Solution and solid state	[81]
		280 nm, 450 nm			Fluorescence spectroscopy	Amyloid fiber suspension	[82]
Aβ(1–40)	DAEFRHDSGYEVHHQKLVFFAEDVGSNKGAIIGLMVGGVV		450 nm, >488 * nm		Time-correlated single-photon-counting fluorescence lifetime imaging	Fibrils in solid state	[27]
			405 nm, >488 * nm		Confocal microscopy	Fibrils in solid state	
		275 nm, 470 * nm			Fluorescence spectroscopy	Fibrils in solution	[58]
		350 nm, 436 nm, and 380 nm, 470 nm			Fluorescence spectroscopy	Fibrils in solution	[83]
		350 nm, 430 nm, and 380 nm, 462 nm			Fluorescence spectroscopy	Fibrils in solution	
Aβ(33–42)	GLMVGGVVIA		450 nm, >488 * nm		Time-correlated single-photon-counting fluorescence lifetime imaging	Fibrils in solid state	[27]
			405 nm, >488 * nm		Confocal microscopy	Fibrils in solid state	
Aβ(30–35)	AIIGLM	320 nm, 420 nm			Fluorescence spectroscopy	Fibrils in solid state	[84]
		310 nm, 425 nm			Fluorescence spectroscopy	Fibrils in solid state	
Aβ(35–42)	MVGGVVIA	365 nm, 470 nm			Fluorescence spectroscopy	Amyloid fiber suspension	[81]
Aβ(16–21)	KLVFFA	359 nm, 461 nm			Fluorescence microscopy	Peptide films	[85]
		330–400 nm, 410–500 nm			Fluorescence spectroscopy	Fibrils in solution and solid state	
Human amylin (1–37) or islet amyloid polypeptide (hIAPP)	KCNTATCATQRLANFLVHSSNNFGAILSSTNVGSNTY			640 nm, 695 nm	Fluorescence spectroscopy	Amyloid fiber suspension	[32]
		280 nm, 382 */450 * nm			Fluorescence spectroscopy	Amyloid fiber suspension	[86]
		325–375 nm, 435–485 nm	450–490 nm, 500–550 * nm		Fluorescence microscopy	Dried aggregates, fibrils in solid state	[87]
		305 nm, 430 * nm	305 nm, 525 * nm		Fluorescence spectroscopy	Fibrils in solution	
		280 nm, 450 nm			Fluorescence spectroscopy	Amyloid fiber suspension	[82]
Aβ(1–42) :hIAPP(1–37) (1:1)	DAEFRHDSGYEVHHQKLVFFAEDVGSNKGAIIGLMVGGVVIA:KC(S-)NTATC(S-)ATQRLANFLVHSSNNFGAILSSTNVGSNTY	280 nm, 450 nm			Fluorescence spectroscopy	Amyloid fiber suspension	[82]
EKKE four-residue peptide	EKKE		405 nm, N.R. **		Confocal microscopy	Amyloid assemblies	[88]
Semaglutide	HAib-EGTFTSDVSSYLEGQAAK(C18diacid-γ-Glu-OEG-OEG)EFIAWLVRGRG	344 nm, 420 nm			Fluorescence spectroscopy	Fibrils in solution	[89]
Ferrocene (Fc)-based peptide	Fc-YYGCGPGRC		490 nm, 520 nm		Fluorescence spectroscopy	Spherical nanoparticle solutions	[90]
Glycyl-L-histidyl-L-lysine-Cu peptide	GHK	375 nm, 435 nm		435 nm, 830 nm	Fluorescence spectroscopy, confocal microscopy	Nanoparticles	[91]
FINYVK peptide	FINYVK	370 nm, 450 * nm			Fluorescence spectroscopy	Amyloid fibers	[92]
fINYVK peptide	fINYVK	370 nm, 450 * nm			Fluorescence spectroscopy	Amyloid fibers	
FiNYVK peptide	FiNYVK	370 nm, 450 * nm	440 nm, 480 * nm		Fluorescence spectroscopy	Amyloid fibers	
FInYVK peptide	FInYVK		440 nm, 480 * nm		Fluorescence spectroscopy	Amyloid fibers	
FINyVK peptide	FINyVK	370 nm, 450 * nm	440 nm, 520 * nm		Fluorescence spectroscopy	Amyloid fibers	
FINYvK peptide	FINYvK	370 nm, 450 * nm	440 nm, 520 * nm		Fluorescence spectroscopy	Amyloid fibers	
FINYVk peptide	FINYVk	370 nm, 450 nm	440 nm, 520 * nm		Fluorescence spectroscopy	Amyloid fibers	
Nucleophosmin-derived peptide NPM1mutA (residues 264–298)	VEAKFINYVKNCFR	336–440 nm, 405–520 nm			Fluorescence spectroscopy	Amyloid fibers	[93]
			440 nm, 460–600 nm		Fluorescence microscopy	Amyloid fibers	
PEG8-F6 peptide	FFFFFF	370 nm, 460 * nm, and 410 nm, 460 * nm			Fluorescence spectroscopy	Amyloid fibers	[75]
FF dipeptide	FF	350 nm, 430 nm			Fluorescence spectroscopy	Fibers	[40]
		255 nm, 350–500 nm			Fluorescence spectroscopy	Microtubes	[94]
FFF tripeptide	FFF	265 nm, 460 nm, and 360 nm, 420–480 nm	410 nm, 480–510 nm		Fluorescence spectroscopy	Irregular nanospheres in solid state	[95]
			420 nm, 520 nm and 502 nm, 550 nm	525 nm, 610 nm	Fluorescence spectroscopy and microscopy	Nanodots suspended in ethylenglicole	[96]
			405 nm, 460 nm		Fluorescence spectroscopy	Fibers	[97]
			405 nm, 560 nm		Fluorescence spectroscopy	Fibers/nanospheres in solid state	[98]
		370 nm, 450 nm	410 nm, 520 nm		Fluorescence spectroscopy	Wafer and film in solid state	[99]
		365–400 nm, 482–484 nm	425–600 nm, 500–620 nm		Fluorescence spectroscopy	Solution of dots	[52]
		365 nm, 425 nm	365 nm, 570 nm		Fluorescence spectroscopy	Solid state	
		360 nm, 440 nm	360 nm, 560 nm		Fluorescence spectroscopy	Solid state tapes	[100]
		Blue, yellow and red			Fluorescence spectroscopy	Nanospheres	[24]
α,β-peptide	Boc-(S-Ala-β-2R,3R-Fpg)3	315 nm, 385 nm			Fluorescence spectroscopy	Amyloid fiber suspension	[101]
Peptide Boc-YW + Zn(II)	Boc-YW	365 nm, 428 nm			Fluorescence spectroscopy and microscopy	Nanoparticles	[102]
Peptide Boc-YW + Zn(II)	Boc-YW	365 nm, 433 nm			Fluorescence spectroscopy and microscopy	Nanoparticles	
Bone Marrow Homing Peptide 1 (BMHP1)-derived peptide	Biotin-GGGAFASAKA	350–500 nm, 450 nm	350–500 nm, 510 nm	350–500 nm, 610–620 nm	Fluorescence spectroscopy	Hydrogel	[103]
LDLK12 peptide variant	(LDLK)3/SSL(LDLK)3/KLP(LDLK)3/tris(LDLK)3	350–500 nm, 450 nm	350–500 nm, 510 nm	350–500 nm, 610–620 nm	Fluorescence spectroscopy	Hydrogel	
PEG-F6 (PEG8, 12, 18, and 24) peptides	PEG-FFFFFF	450 nm, 460–480 nm	520 nm, 530–560 nm	635, 645–650 nm	Fluorescence microscopy	Peptide films and aggregates	[104]
PEG8-F6 peptide	PEG8-FFFFFF	370 nm, 460 * nm, and 410 nm, 460 * nm			Fluorescence spectroscopy	Amyloid fibers	[75]
		405 nm, 490–510 nm			Fluorescence confocal microscopy	Amyloid fibers	
PEG12-F6 peptide	PEG12-FFFFFF	370 nm, 460 * nm 410 nm, 460 * nm			Fluorescence spectroscopy	Amyloid fibers	[76]
		405 nm, 490–510 nm			Fluorescence and confocal microscopy	Amyloid fibers	
PEG18-F6 peptide	PEG18-FFFFFF	370 nm, 460 nm, and 410 nm, 460 nm			Fluorescence spectroscopy	Amyloid fibers	[76]
		405 nm, 490–510 * nm			Fluorescence microscopy	Amyloid fibers	
PEG24-F6 peptide	PEG24-FFFFFF	370 nm, 460 * nm	460 nm, 530 * nm		Fluorescence spectroscopy	Amyloid fibers	[76]
		359 nm, 461 * nm	488 nm, 507 * nm	555 nm, 580 * nm	Fluorescence microscopy	Amyloid fibers	
PEG2-F2 peptide	PEG2-FF	340–420 nm, 405–480 nm			Fluorescence spectroscopy	Amyloid fiber suspension	[36]
PEG6-F2 peptide	PEG6-FF	370–470 nm, 460–505 nm			Fluorescence spectroscopy	Amyloid fiber suspension	
F6 peptide	FFFFFF	359 nm, 461 nm, and 330–430 nm, 400–490 nm	488 nm, 507 nm		Fluorescence microscopy	Peptide films	[85]
Dip-Dip peptide	(β,β-diphenyl-A)_2_	280–410 nm, 415/475 nm			Fluorescence spectroscopy	Layered needle-like tubular structures	[105]
Boc-Dip-Dip peptide	(Boc-β,β-diphenyl-A)_2_	370 nm, 411/436/462 nm			Fluorescence spectroscopy	Layered needle-like tubular structures	[105]
Trp-Phe peptide	WF	280 nm, 390/423 nm			Fluorescence spectroscopy	Nanoparticles	[106]
Peptide fragments derived from hydrolysis of β-LG	N.R. **	375 nm, 460 nm			Fluorescence spectroscopy	Fibers	[107]
ε-PLL peptide	ε-poly-K	336 nm, 407 nm			Fluorescence spectroscopy	Solution	[74]
PLL-OL peptide	α-poly-K sodium oleate	310–400 nm, 380–475 nm			Fluorescence spectroscopy	Vesicle suspension	[108]
		300–380 nm, 462 nm			Fluorescence spectroscopy	Film	
PBLG peptide	poly(γ-benzyl-glutamate)	260–400 nm, 440 nm			Fluorescence spectroscopy	Solution	[109]
PLGA peptide	poly-E	260–400 nm, 440 nm			Fluorescence spectroscopy	Solution	[109]
PLGA-Na peptide	poly-E sodium salt	260–400 nm, 440 nm			Fluorescence spectroscopy	Solution	[109]
Alanine oligopeptide (OALA)	AAAAA	325 nm, 400 nm			Fluorescence spectroscopy and microscopy	Solid state	[110]
Valine oligopeptide (OVAL)	VVVVV	325 nm, 400 nm			Fluorescence spectroscopy and microscopy	Solid state	[110]
Isoleucine oligopeptide (OILE)	IIIII	325 nm, 400 nm			Fluorescence spectroscopy and microscopy	Solid state	[110]
Alanine polypeptide (PALA-HT)	poly-A	325 nm, 445nm			Fluorescence spectroscopy and microscopy	Solid state	[110]
Alanine polypeptide (PALA-SS)	Guanidin-poly-A	325 nm, 445 nm			Fluorescence spectroscopy and microscopy	Solid state	[110]

* Data inferred from the figures of the related reference. ** Not reported (N.R.).

**Table 3:** Intrinsic visible fluorescence emission detected in amyloids formed by peptide nucleic acid (PNA) diphenylalanine (FF) conjugates x-FF, xx-FF, and FF-xx, where x = adenine (a), thymine (t), guanine (g), or cytosine (c). Amino acids are reported using one-letter code.

System	Excitation, Emission Wavelength/Range (λexc, λem)			Methodology Used for the Experiment	Physical State of the Sample	Ref.
	UV–Blue	Green	Red–NIR			
c-FF	359 nm, 461 * nm	488 nm, 507 * nm		Fluorescence microscopy	Crystalline form	[77]
	310 nm, 380 * nm			Fluorescence spectroscopy	Solution	
g-FF	359 nm, 461 * nm	488 nm, 507 * nm		Fluorescence microscopy	Crystalline form	[77]
	310 nm, 375 * nm			Fluorescence spectroscopy	Solution	
a-FF	359 nm, 461 * nm	488 nm, 507 * nm		Fluorescence microscopy	Crystalline form	[77]
	320 nm, 410 * nm			Fluorescence spectroscopy	Solution	
t-FF	359 nm, 461 * nm	488 nm, 507 * nm		Fluorescence microscopy	Crystalline form	[77]
	320 nm, 390 * nm			Fluorescence spectroscopy	Solution	
cc-FF	359 nm, 461 * nm	488 nm, 507 * nm		Fluorescence microscopy	Crystalline form	[77]
	320 nm, 364 * nm			Fluorescence spectroscopy	Solution	
gg-FF	359 nm, 461 * nm	488 nm, 507 * nm		Fluorescence microscopy	Crystalline form	[77]
	310 nm, 375 * nm			Fluorescence spectroscopy	Solution	
aa-FF	359 nm, 461 * nm	488 nm, 507 * nm		Fluorescence microscopy	Crystalline form	[77]
	340 nm, 420 * nm			Fluorescence spectroscopy	Solution	
tt-FF	359 nm, 461 * nm	488 nm, 507 nm		Fluorescence microscopy	Crystalline form	[77]
	330 nm, 390 * nm			Fluorescence spectroscopy	Solution	
FF-gc	340 nm, 460 nm			Fluorescence spectroscopy	Solution	[111]
	359 nm, 461 nm	488 nm, 507 nm	555 nm, 580 nm	Fluorescence microscopy	Solid state	
gc-FF	340 nm, 400 nm			Fluorescence spectroscopy	Solution	[111]
	359 nm, 461 nm	488 nm, 507 nm	555 nm, 580 nm	Fluorescence microscopy	Solid state	
FF-gc	340 nm, 460 nm			Fluorescence spectroscopy	Solution	[111]
	359 nm, 461 nm	488 nm, 507 nm	555 nm, 580 nm	Fluorescence microscopy	Solid state	
gc-FF	310 nm, 380 nm			Fluorescence spectroscopy	Solution	[111]
	359 nm, 461 nm	488 nm, 507 nm	555 nm, 580 nm	Fluorescence microscopy	Solid state	[111]

* Data inferred from the figures of the related reference.

#### 3.2.2. Cyclic Dipeptides

Although the backbone of cyclic dipeptides cannot precisely emulate the network of hydrogen bonds that stabilize the β-structure of the cross-β motif (see Figure 1), they present a remarkable propensity to form supramolecular assemblies that are also endowed with special spectroscopic properties.

The initial evidence on the ability of cyclic dipeptides to form fluorescent aggregates able to emit in the visible region of the spectrum was obtained by Gazit and Rosenman et al. [112]. Upon heating the linear FF dipeptide, they generated blue fluorescent aggregates that were actually formed by cyclo-FF (see Table 4). The cyclization of other blue fluorescent dipeptides formed by aliphatic residues such as cyclo-LL [95,113] confirmed that, in line with the results obtained for linear peptides, the presence in the sequence of aromatic residues was not necessary to generate the peculiar spectroscopic properties reviewed here. Moreover, different studies have shown that cyclic peptides may self-assemble into fluorescent nanostructures that are different from their linear counterparts [114,115].

More recently, it has been reported that the morphology and the properties of the aggregates formed by cyclo-dipeptides may be easily changed via chemical (amino acid replacement and conjugation) or physical (co-assembly) modifications [116]. The ad hoc modifications and derivatization of these cyclic dipeptides, as well as those based on the Trp residue, make the fluorescence emission tunable from the visible into the NIR spectral region.

**Table 4:** Intrinsic visible fluorescence emission detected in amyloids formed by cyclic peptides. Amino acids are reported using one-letter code.

Peptide System	Excitation, Emission Wavelength/Range (λexc, λem)		Methodology Used for the Experiment	Physical State of the Sample	Ref.
	UV–Blue	Green			
Cyclo-FW	370 nm, 460 nm		Fluorescence spectroscopy	Needle-like crystals	[116]
Cyclo-WW	425 nm, 520 nm		Fluorescence spectroscopy	Spherical nanoparticles	[116]
Cyclo-FW	370 nm, 430 nm		Fluorescence spectroscopy	Multibranched nanoflower	[116]
Cyclo-HH	370 nm, 480 nm		Fluorescence spectroscopy	Nanofibers	[116]
Cyclo-YY		480 nm, 570 nm	Fluorescence spectroscopy	Nanorods	[116]
Cyclo-FF		450 nm, 530 nm	Fluorescence spectroscopy	Spherical nanoparticles	[116]
Cyclo-GW	300 nm, 420 nm, and 400 nm, 440 nm		Fluorescence spectroscopy	Monoclinic needle-like crystals	[117]
Cyclo-Dip-Dip	280–410 nm, 415–475 nm		Fluorescence spectroscopy	Layered needle-like tubular structures	[105]
Cyclo-FF	260 nm, 305 nm, and 370 nm, 450 nm	305 nm, 400–500 nm	Fluorescence spectroscopy	Nanotubes in solid state on quartz	[112]
	265 nm, 300 nm		Fluorescence spectroscopy and microscopy	Coating of nanotubes in solid state on silicon	[115]
	265 nm, 300–440 nm		Fluorescence spectroscopy and microscopy	Peptide films and coating of nanotubes in solid state on silicon	
	370 nm, 420–460 nm		Fluorescence spectroscopy	Nanofiber in solution	[113]
	265 nm, 460 nm, and 360 nm, 420–460 nm		Fluorescence spectroscopy	Fibers in solid state	[95]
		380 nm, 620 nm	Fluorescence spectroscopy, scanning near-field optical microscopy	Platelet solid state	[118]
Cyclo-LL	370 nm, 420–460 nm		Fluorescence spectroscopy	Nanofibers in solution	[113]
	360 nm, 420–460 nm		Fluorescence spectroscopy	Ultrathin nanowires	[95]

## 4. Intrinsic Visible Fluorescence Emitted by Individual Amino Acids and Their Variants

The first evidence that individual amino acids can emit visible fluorescence dates back to 2001 when Homchaudhuri and Swaminathan [28,29] demonstrated that Lys can emit blue fluorescence (~435 nm) upon excitation at 355 nm. However, despite this sporadic observation, systematic analyses of the fluorescence properties of individual amino acids have been strongly stimulated by the observation that self-assembled proteins and peptides can emit in the visible regions of the spectrum [119]. In recent years, distinct reports have confirmed the general ability of amino acid aggregates to emit visible fluorescence [40,74] (Table 5). In line with the results obtained for proteins and peptides, this spectroscopic property was detected for both aromatic and non-aromatic amino acids [74]. Moreover, the ability of some amino acids such as Ser [74,120,121] and Lys [74] to emit green fluorescence has been demonstrated. It has also been demonstrated that aggregates formed by His are promising candidates for the development of bio-photonic devices [120]. Interestingly, it has been shown that the transformation of Glu to pyroglutamine is associated with a remarkable enhancement of the fluorescence emission that was ascribed to the formation of particularly short and strong hydrogen bonds [122]. Very recently, a systematic analysis of the spectroscopic properties of amino acids has provided interesting information on the impact that molecular packing and chemical characteristics have on this phenomenon [121]. The dependence of the fluorescence emission, for some amino acids such as Cys, on specific crystal packings has also suggested the possibility of reversibly regulating these optically active states.

**Table 5:** Individual amino acids emitting visible fluorescence. Amino acids are reported using one-letter code.

System	Excitation, Emission Wavelength/Range (λexc, λem)		Methodology Used for the Experiment	Physical State of the Sample	Ref.
	UV–Blue	Green			
H	360 nm, 424/445 nm, and 380 nm, 450 nm, and 400 nm, 452–500 nm	405 nm, 488 nm, and 420 nm, 500 nm, and 440 nm, 515 nm, and 460 nm, 550 nm, and 480 nm, 560 nm	Fluorescence spectroscopy and confocal microscopy	Microplates	[120]
Q	360 nm, 430 nm		Fluorescence spectroscopy	Solid state	[122]
W	350 nm, 400/450 nm		Emission-excitation matrices (EEMs)	Solution	[40]
Y	350 nm, 400/440 nm		Emission-excitation matrices (EEMs)	Solution	[40]
pyro-Q	340 nm, 420 nm		Fluorescence spectroscopy	Solid state	[122]
Amino acid crystals	405 nm, N.R. **		Confocal spectroscopy	Crystals	[121]
Ornitin	405 nm, N.R. **		Confocal spectroscopy	Crystals	[121]
2,4-diaminobutyric acid	405 nm, N.R. **		Confocal spectroscopy	Crystals	[121]
D-2,3-diaminopropionic acid	405 nm, N.R. **		Confocal spectroscopy	Crystals	[121]
F	370 nm, 452 nm, and 380 nm, 454 nm, and 390 nm, 456 nm	400 nm, 458 nm, and 410 nm, 500 nm, and 420 nm, 500–540 nm	Fluorescence spectroscopy	Fibrillar solution	[121]
	350 nm, 400/430 nm		Emission-excitation matrices	Solution	[40]
K	340 nm, 433 nm 365 nm, and 440/488 nm	460 nm, 511 nm	Fluorescence spectroscopy	Concentrated solution	[74]
K	300–365 nm, 385–420 nm	365 nm, 524 nm	Fluorescence spectroscopy	Recrystallized solids	
S	320–440 nm, 385–484 nm	320 nm, 513 nm, and 440 nm, 484/598 nm	Fluorescence spectroscopy	Recrystallized solids	
S	312 nm, 427 nm	480 nm, 529 nm	Fluorescence spectroscopy	Concentrated solution	
I	320–440 nm, 384–484 nm	320 nm, 507 nm, and 440 nm, 484/598 nm	Fluorescence spectroscopy	Recrystallized solids	
N-acetyl-A	260–400 nm, 440 nm		Fluorescence spectroscopy	Solution	[109]
Amyloid metabolites (W Y, and F)	405 nm, 450 nm		Fluorescence spectroscopy	Solution	[123]
GlyBA glycine catalyzed by boric acid (G-BA)	452 nm, 460 nm		Fluorescence spectroscopy	Carbonaceous structures	[124]
Phe dimer assembly (F-DA)	370 nm, 425 nm		Fluorescence spectroscopy and microscopy	Amyloid fibers/ hydrogel	[125]

** Not reported (N.R.).

## 5. Open Issues: The Electronic Basis of the Fluorescence Emission and Its In Vivo Applications

The data reported in the previous sections demonstrate the wide interest in this peculiar spectroscopic that impacts rather different scientific areas. Notwithstanding the remarkable efforts to characterize and develop peptide-based assemblies that can emit fluorescence in the visible region of the electromagnetic spectrum, some crucial issues deserve special efforts. Among others, the definition of the electronic basis of this fluorescence and the limitations of the in vivo applications caused by the low-intensity fluorescence of the available systems represent topics for which progress is particularly needed.

Typical protein fluorescence that is characterized by emission up to 360 nm is caused by the excitation of the delocalized electrons present in the aromatic ring present in the side chains of residues such as Trp, Tyr, and Phe [126]. The fluorescence described here must have a radically different origin. However, despite the overwhelming evidence of the ability of self-assembled proteins and peptides to emit visible fluorescence, the structural and electronic basis underlying this puzzling phenomenon is far from being elucidated. Indeed, although in some specific cases the anomalous visible emission of proteins has been attributed to the oxidation of specific residues such as Tyr [58], general theories that explain the structure-based amyloid emission are still missing. Nevertheless, in the last few years, interesting suggestions have been proposed. Indeed, several independent computational approaches have highlighted the role of specific factors, which, alone or in combination, may account for the experimental observation. One of the first attempts to explain fluorescence emission by amyloids formed by non-aromatic residues was made by Kaminski Schierle and coworkers [81]. By performing ab initio molecular dynamics simulations, these authors highlighted the importance that proton transfer across hydrogen bonds could have in this process. They also suggested that proton delocalization between the charged ends of the chains could play a significant role. This suggestion, however, was not corroborated by a systematic analysis of the fluorescence emission of hexa-phenylalanine variants endowed with different charged states, which all presented similar spectroscopic properties [85]. More recently, it has been proposed that the deplanarization of the amide groups makes possible the excitation in the near-UV. Subsequently, the relaxation of the photoexcited peptides may generate green fluorescence [127]. By assuming that this fluorescence emission may have a common origin, independently of the molecular complexity of the self-assembled systems, it is relevant to mention that the fluorescence emission generated by the transformation, upon heating, of Glu to pyroglutamine is due to the formation of very strong hydrogen bonds characterized by a length that is as low as 2.45 Å [122].

Very recently, a new intriguing hypothesis has been proposed [128]. To explain the origin of the fluorescence emission by compounds that do not contain either aromatic residues or conjugated systems, in this study Miran and coworkers suggested that carbonyl elongation is an important vibrational event that favors non-radiative decay toward the ground state. The blocking of this motion in highly restricted systems originates the fluorescence emission. The generality of this interpretative model, which is based on the properties of the carbonyl group, an omnipresent moiety in these emitting systems, makes it particularly appealing.

It is important to note that a full understanding of this phenomenon is of crucial importance for designing new bioinspired materials endowed with high-efficiency fluorescence [129] and for developing better devices and approaches for their sensing in vivo. Although the present review has focused on the in vitro characterization of this phenomenon, its potential for in vivo applications has been investigated in several papers in the literature [32,54,123,130,131,132,133,134,135].

## 6. Conclusions

A remarkable number of studies in the literature have described a peculiar fluorescence emission in the visible region of the electromagnetic spectrum by amyloids formed by either small peptides or large proteins. Although the chemical–physical basis of this phenomenon is still highly debated, it has attracted the attention of the scientific community for its many implications in both basic and applied science. Intriguingly, recent studies have suggested that this peculiar spectroscopic property can also be detected in other systems such as individual amino acids and metabolites in highly restricted environments. Although many interesting reviews have focused on this topic, to the best of our knowledge, no attempts to systematically record and classify self-assembled proteins, peptides, and individual amino acids have been hitherto reported. Although the collection of exhaustive data from the literature is made difficult by the different terminology associated with the same phenomenon in different scientific areas, the survey reported here suggests that the specific self-assembly, such as the cross-β motif that characterizes amyloids, is likely sufficient to generate this intriguing phenomenon. This idea could be further verified if in future studies the ability of amyloids to emit fluorescence as a consequence of the binding to specific probes such as ThT and/or Congo Red will be compared to the fluorescence emission of the intrinsic label-free system.

The data collected here also show that the intrinsic fluorescence exhibited by self-assembling proteins/peptides is a multifaceted phenomenon in which different systems may exhibit fluorescence in different spectral regions. Indeed, most of the systems present emission in the blue region, whereas green fluorescence is rare. Although the analysis of these variations in a common theme may help to unveil the structural and chemical–physical basis underlying this phenomenon, in many studies the characterization of the spectroscopic properties is limited to specific wavelength windows. It is also important to mention that, in recent years, the atomic-level characterization of amyloids has made impressive advances even for highly complex systems [8]. However, structural studies are rarely coupled with spectroscopy characterizations, although this could be of extreme importance for unraveling correlations between structural properties, for example, the parallel or antiparallel association of the strands in the cross-β beta motifs, and the type of fluorescence emission. This type of information could also be important to elaborate upon new interpretative mechanisms of the phenomenon or to validate the existing ones. As discussed above, the definition of the basis of this fluorescence emission is a topic whose implications go well beyond basic science. Indeed, a deeper understanding of this phenomenon is of crucial importance for designing new biomaterials endowed with a better quantum yield compared to the existing assemblies, as this limitation represents a major obstacle for practical applications [129]. A full understating of this puzzling phenomenon may also help to exploit this property of cross-β assemblies in the in vivo tracing of amyloid formation, as intrinsic fluorescence may be a complementary or alternative option to the use of external fluorophores [136,137,138], thus avoiding any risk of perturbations induced by modifications during aggregation [27].

## Supplementary Materials

The supporting information can be downloaded at https://www.mdpi.com/article/10.3390/ijms24098372/s1.
