# Supplementary material for: A Comprehensive Analysis of the Intrinsic Visible Fluorescence Emitted by Peptide/Protein Amyloid-like Assemblies
Source: Int J Mol Sci. 2023 May 6;24(9):8372. doi: 10.3390/ijms24098372 (PMC10178990; doi:10.3390/ijms24098372)
Supplement: Supplementary file 1 [file ijms-24-08372-s001.zip › ijms-2349960-Supplementary materials.pdf]

Supplementary materials

A Comprehensive Analysis of the Intrinsic Visible Fluorescence Emitted by Peptide/Protein Amyloid-like Assemblies

Nicole Balasco [1], Carlo Diaferia [2], Elisabetta Rosa [2], Alessandra Monti [3], Menotti Ruvo [3], Nunzianna Doti [3,*] and Luigi Vitagliano [3,*]

[1] Institute of Molecular Biology and Pathology, National Research Council (CNR), Piazzale Aldo Moro 5, 00185 Rome, Italy; nicole.balasco@cnr.it
[2] Department of Pharmacy and CIRPeB, Research Centre on Bioactive Peptides "Carlo Pedone", University of Naples "Federico II", Via Montesano 49, 80131 Naples, Italy; carlo.diaferia@unina.it (C.D.); elisabetta.rosa@unina.it (E.R.)
[3] Institute of Biostructures and Bioimaging (IBB), National Research Council (CNR), 80131 Napoli, Italy; alessandra.monti@ibb.cnr.it (A.M.); menotti.ruvo@unina.it (M.R.)
* Correspondence: nunzianna.doti@cnr.it (N.D.); luigi.vitagliano@unina.it (L.V.)

Table S1. Literature survey protocol and outcome.

PUBMED QUERY	TOTAL PAPERS	PAPERS OF INTEREST BASED ON THE ABSTRACT
amyloid "intrinsic fluorescence"	142	33
amyloid peptide visible luminescence	15	7
amyloid protein visible luminescence	20	1
amyloid photoluminescence	27	6
amyloid label-free fluorescence	54	12
amyloid autofluorescence	95	28
